# Involvement of mTOR Pathways in Recovery from Spinal Cord Injury by Modulation of Autophagy and Immune Response

**DOI:** 10.3390/biomedicines9060593

**Published:** 2021-05-24

**Authors:** Ingrid Vargova, Lucia Machova Urdzikova, Kristyna Karova, Barbora Smejkalova, Tolga Sursal, Veronika Cimermanova, Karolina Turnovcova, Chirag D. Gandhi, Meena Jhanwar-Uniyal, Pavla Jendelova

**Affiliations:** 1 Institute of Experimental Medicine, Academy of Sciences of the Czech Republic, Videnska, 1083, 142 20 Prague, Czech Republic; ingrid.vargova@iem.cas.cz (I.V.); lucia.machova@iem.cas.cz (L.M.U.); kristyna.karova@iem.cas.cz (K.K.); barbora.smejkalova@iem.cas.cz (B.S.); ve.cimermanova@gmail.com (V.C.); karolina.turnovcova@iem.cas.cz (K.T.); 2 2nd Faculty of Medicine, Charles University, V Uvalu 84, 150 06 Prague, Czech Republic; 3 Department of Neurosurgery, Westchester Medical Center, New York Medical College, Valhalla, NY 10595, USA; tolga.sursal@wmchealth.org (T.S.); chirag.gandhi@wmchealth.org (C.D.G.)

**Keywords:** spinal cord injury, mTOR, rapamycin, pp242, dual inhibition, autophagy, inflammation

## Abstract

Traumatic spinal cord injury (SCI) is untreatable and remains the leading cause of disability. Neuroprotection and recovery after SCI can be partially achieved by rapamycin (RAPA) treatment, an inhibitor of mTORC1, complex 1 of the mammalian target of rapamycin (mTOR) pathway. However, mechanisms regulated by the mTOR pathway are not only controlled by mTORC1, but also by a second mTOR complex (mTORC2). Second-generation inhibitor, pp242, inhibits both mTORC1 and mtORC2, which led us to explore its therapeutic potential after SCI and compare it to RAPA treatment. In a rat balloon-compression model of SCI, the effect of daily RAPA (5 mg/kg; IP) and pp242 (5 mg/kg; IP) treatment on inflammatory responses and autophagy was observed. We demonstrated inhibition of the mTOR pathway after SCI through analysis of p-S6, p-Akt, and p-4E-BP1 levels. Several proinflammatory cytokines were elevated in pp242-treated rats, while RAPA treatment led to a decrease in proinflammatory cytokines. Both RAPA and pp242 treatments caused an upregulation of LC3B and led to improved functional and structural recovery in acute SCI compared to the controls, however, a greater axonal sprouting was seen following RAPA treatment. These results suggest that dual mTOR inhibition by pp242 after SCI induces distinct mechanisms and leads to recovery somewhat inferior to that following RAPA treatment.

## 1. Introduction

Spinal cord injury (SCI) is one of the most frequent causes of disability. Traumatic SCI is characterized as a permanent or temporary loss of function of the spinal cord that is usually caused by physical impact on the spine that dislocates or fractures vertebrae [1,2]. After the primary mechanical injury to the tissue, a secondary inflammatory cascade is initiated that extends from a few hours to a few weeks after the injury. This sub-acute phase includes mechanisms such as oxidative stress, mitochondrial dysfunction, decreased ATP production, activation of astrocytes and microglia, immune cell invasion, the release of cytokines, mediated cell death, and others [3]. Although the initial inflammatory cellular response to SCI is supposed to contain the damage to the injury site, it can also progress into a succession of events leading to additional injury and hindering later attempts of regeneration [4]. The secondary injury can be detrimental; however, treatments that target the underlying cause may lessen the level of injury and improve the degree of recovery. Anti-inflammatory therapies at early stages of SCI have been studied in recent years. Applications of compounds, including riluzole [5], fingolimod [6], and fibroblast growth factor [7], or naturally-occurring anti-inflammatory compounds such as curcumin [8] and the polyphenol epigallocatechin-3-gallate (EGCG) [9] have shown limited, but promising results in recovery from SCI.

One of the cellular mechanisms disrupted after SCI that contribute to secondary injury is the inhibition of autophagic flux. Autophagy is an adaptive catabolic process that occurs as a reaction of the cell to various types of stress, such as growth factor depletion, infection, and nutrient or oxygen deficiency. The main function of autophagy is to provide substrates for essential cellular functions during stress; however, it also has a major cytoprotective role in selective elimination of harmful cytosolic material [10]. Neurons rely heavily on autophagy, which is evident from the vulnerability of the brain to mutations of genes connected to intracellular trafficking, which are strongly linked to neurodegenerative disorders [11]. Autophagy-deficient mice develop PD-like neurodegenerative pathologies, suffer neuronal loss, and eventually die [12,13]. Autophagy modulation has been explored in various diseases [14], especially its induction by rapamycin (RAPA). Given that RAPA penetrates the blood–brain barrier poorly, novel inducers of autophagy such as aromatic carbamates have been proposed as more potent and suitable inducers of autophagy in neurodegenerative diseases [15]. Although it was previously found that autophagy markers increase in the spinal cord after trauma [16], it was shown that this upregulation is caused by the pathological accumulation of autophagosomes and disrupted autophagic flux [17]. Restoration of proper autophagic flux by overexpression of ATG5 in injured motoneurons was indeed neuroprotective [18]. Modulation of autophagy is, therefore, a potential treatment of SCI, as shown in several studies with positive outcomes [19,20].

Mammalian target of rapamycin (mTOR) is a serine/threonine protein kinase that has a central role in the regulation of cell metabolism, death, survival, and proliferation through its involvement in mechanisms such as mRNA transcription, translation, apoptosis, and autophagy [21]. mTOR has been recognized as a major negative regulator of autophagy through multiple pathways [22,23,24]. Inhibition of mTOR by RAPA has led to an upregulation of autophagy and a neuroprotective effect in multiple neurodegenerative disorders [25,26], including SCI [19,20,27]. The major role of the mTOR pathway is its function as a regulator of the immune response. RAPA is a potent immunosuppressant and has been previously shown to reduce neuroinflammation [28,29], as well as apoptotic cell death [30]. These findings indicate that inhibition of the mTOR pathway can be protective against secondary damage in SCI through induction of autophagy, in addition to neuroinflammation containment.

mTOR functions through two functionally and structurally similar, but different complexes, mTOR complex 1 (mTORC1) and mTOR complex 2 (mTORC2) [31]. Both complexes are a part of the PI3K/Akt/mTOR pathway but are distinguished by their sensitivity to RAPA, which exclusively inhibits mTORC1 activity. mTORC2 responds only to long-term inhibition by RAPA [32]. A new generation of mTOR inhibitor, pp242, has been shown to inhibit both mTORC1 and mTORC2 [33], as well as induce autophagy and apoptosis in vitro [34]. Dual inhibition of mTORC1/2 with pp242, therefore, has the potential to more significantly alter the sub-acute phase of SCI compared to RAPA.

In this study, we investigated the effects of two types of mTOR inhibitors, RAPA and pp242, on the mTOR signaling pathway as it relates to autophagy, inflammation, and recovery in rats after SCI.

## 2. Materials and Methods

### 2.1. Animals

Male Wistar rats (*n* = 36), with an average weight of 300–330 g, were used in this study. The number of animals included in the study was reduced as much as statistically feasible for each experiment, in accordance with the European Communities Council Directive of 22 September 2010 (210/63/EU). The experimental procedures were approved by the Ethics Committee of the Institute of Experimental Medicine CAS and Ethics Committee of the Czech Academy of Sciences under project No. 55/2017.

### 2.2. Spinal Cord Injury

Rats were subjected to surgery to induce SCI using a previously described balloon-compression model of SCI [35]. Briefly, dorsal laminectomy of the T10 vertebrae was conducted on anesthetized animals (isoflurane 2.5–3 vol.%, Aerrane; buprenorphine, 0.05–0.1 mg/kg subcutaneous Vetergesic Multidose, Reckitt Benckiser, Slough, UK). Next, a Fogarty catheter (2 French, Edwards Lifesciences, Irvine, CA, USA) was inserted into the epidural cavity, positioning the balloon at the level of the T8 vertebral body. The balloon was filled with 15 µL of saline solution, which compressed the spinal cord. After 5 min, the balloon was extracted, and the injury site was sutured. The body temperature of the animal during the operation was maintained using a heating pad [36]. During recovery, ampicillin (60 mg/kg) was administered daily for 5 days postoperatively, and the rats’ pain was reduced by administering buprenorphine (Vetergesic Multidose for cats and dogs, 0.05–0.1 mg/kg), as required. Animals were aided manually with urinary excretion and feeding.

Rats were randomized into four groups postoperatively: RAPA (5 mg/kg, ChemScience, Quebec, CA), pp242 (5 mg/kg, APExBIO, Huston, TX, USA), vehicle-treated SCI control group (2% DMSO, 5% triton, saline), and a no-lesion and no-treatment control group (Table 1). Dosages were chosen on the basis of previous animal studies using RAPA [37] and pp242 [38] treatment. The vehicle solution was chosen according to pp242 solubility, according to the manufacturer’s recommendations with modification. The treatment was intraperitoneally administered daily, starting from the second day and repeated until the sixth day after the SCI (five treatments total), after which the rats were sacrificed on day 7 after surgery.

### 2.3. Western Blot Analysis

Rats were sacrificed, as described above, and spinal cords were rapidly removed and stored at −80 °C until protein isolation. Tissue segments (~1 cm) of the injured spinal cord were cut and homogenized in RIPA buffer (50 mM Tris (pH 8), 150 mM NaCl, 1% Triton X-100, 1 mM EDTA, 0.5% sodium deoxycholate, 0.1% sodium dodecyl sulfate, a protease inhibitor (PhosphoSTOP™ EASYpack, Roche, Sigma, St. Louis, MO, USA), and phosphatase inhibitor (cOmplete™ Protease Inhibitor Cocktail, Roche, Sigma). Samples were kept on ice for 30 min and vortexed every 5 min. After that, samples were sonicated in three pulse bursts for 10 s and vortexed again, before centrifuging at 14,000× *g* at 4 °C for 15 min. Protein concentration in the supernatant was measured using a BCA assay (Pierce). Protein separation was performed using 25 μg on protein on 4–15% gradient Mini-PROTEAN TGX Stain-free gels (Bio-Rad, Hercules, CA, USA). After protein separation, the gel was activated for 2 min using a 302 nm UV lamp and captured on an Azure c400 imaging system. Protein transfer was done using a wet blot on a PVDF membrane (Life Technologies, Carlsbad, CA, USA) and confirmed by visualizing the fluorescent proteins on the membrane using the Azure c400 imager. The blots were blocked using either 5% bovine serum albumin (Sigma, St. Louis, MO, USA) or 5% dried milk (Cell Signaling Technology, Danvers, MA, USA) in Tris-buffered saline/Tween-20 (TBST), depending on individual antibody requirements. Membranes were then incubated with primary antibodies in blocking solution overnight at 4 °C, washed with TBST, and subsequently incubated with secondary antibody in TBST. Antibodies used against specific proteins were as followed: Akt (#4691S; Cell Signaling Technology), phospho-Akt (Ser473) (1:2000; #4060; Cell Signaling Technology), eukaryotic translation initiation factor 4E-binding protein 1 (4E-BP1) (#9644; Cell Signaling Technology), phospho-4E-BP1 (Thr37/46) (#2855; Cell Signaling Technology), microtubule-associated proteins 1A/1B light chain 3B (LC3B) (#2775; Cell Signaling Technology), peroxidase goat anti-mouse secondary antibody (#115-035-003; J.ImmunoResearch, Cabridgeshire, UK), and peroxidase goat anti-rabbit secondary antibody (#111-035-003; J.ImmunoResearch). All primary antibodies were used at the concentration of 1:1000 except where indicated otherwise, and secondary antibodies were used at the concentration of 1:10,000. Secondary antibodies were conjugated with horseradish peroxidase (HRP), and protein bands were visualized using chemiluminescence induced by the ClarifyTM Western ECL Substrate (Bio-Rad) and imaged using Azure Biosystems c400. The relative signal intensity was quantified using Fiji software and normalized relative to total protein content from stain-free signals, which were previously proven to be reliable loading controls [39].

### 2.4. Immunohistochemistry

**Figure A1:**
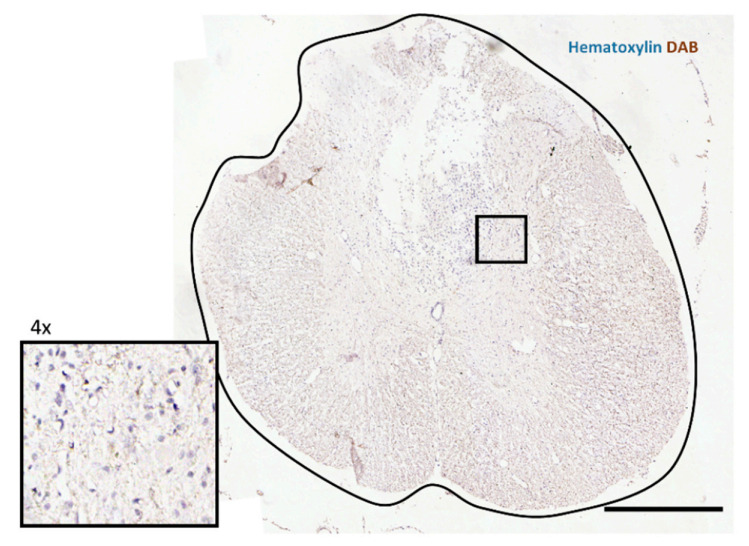
Negative control of immunohistochemical staining. Section of spinal cord with lesion was stained according to the protocol described in Section 2, while leaving out the primary antibody. Scale bar: 500 µm.

**Figure A2:**
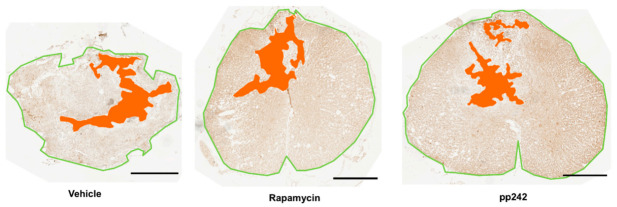
Representative images of lesion size evaluation. Orange areas represent the lesions, while green lines enclose the spinal cord section. Scale bars: 500 µm.

Immunohistochemical analysis included three groups: RAPA (*n* = 5), pp242 (*n* = 5), and vehicle control (*n* = 5) groups. Transcardial perfusion with 4% paraformaldehyde (PFA) in phosphate buffer was performed in all animals at 7 days post SCI. The spinal column was removed and kept in 4% PFA overnight for fixation, after which the sample was dissected, and the spinal cord was extracted. The spinal cord tissue proximal to the injury site (1 cm rostrally and caudally from the lesion center) was kept in sterile phosphate buffer until further use. Tissue sections were embedded in paraffin and cut transversely into 5 μm thick slices. Five slices positioned 1 mm apart from each other along the rostro-caudal axis were incubated with primary antibodies against phosphorylated ribosomal protein S6 (p-S6) (1:150; #4858, Cell Signaling), LC3B (1:100; #2775; Cell Signaling), followed by biotinylated anti-rabbit secondary antibody made in goat (1:400; #BA-1000, Vectorlabs, Burlingame, CA, USA). Detection was done using a VECTASTAIN^®^ ABC Kit (#PK-4005, Vectorlabs), which is based on precipitate formation through HRP reacting with 2,3′-diaminobenzidine (DAB) in the presence of H_2_O_2_. Lastly, cell nuclei were visualized using hematoxylin staining. Figure A1 (Appendix A) shows an image of the negative control (omitting primary antibody). Axonal sprouting was measured by staining the sections with primary antibody against growth-associated protein 43 (GAP43; 1:500, #sc-7457, Santa Cruz Biotechnology, Santa Cruz, CA, USA), followed by secondary antibody conjugated with Alexa-Fluor 488 (1: 200, #A-11029, Thermofisher, Waltham, MA, USA). Whole transversal sections were captured on a LEICA CTR 6500 microscope with FAXS 4.2.6245.1020 (TissueGnostics, Vienna, AT) software. Images were analyzed, and the number of p-S6- or LC3B-positive cells per mm^2^ of tissue was determined using HistoQuest 4.0.4.0154 (TissueGnostics) software. GAP43 and lesion size analysis was done using Fiji software [40]. Images used for lesion analysis were the same as those used for LC3B examination. Lesions were considered parts of the stained spinal cord sections that contained no visible cells stained by hematoxylin (Figure A2, Appendix A). Sections that were too fractured to distinguish lesions from mechanical damage sustained during staining and handling were excluded from analysis. Lesion area was related to the size of the presumed area of the spinal cord section.

### 2.5. Cytokines

The levels of secreted cytokines produced in spinal cord tissue after SCI and subsequent treatment (RAPA, *n* = 6; pp242, *n* = 6; vehicle control, *n* = 7) were measured as described previously [41]. Rats were sacrificed, and a 2 mm portion of the spinal cord at the lesion site was rapidly dissected. Tissue was then incubated for 24 h in a cell culture medium (DMEM, Sigma; 10% FBS; 0.2% primocin). The medium was then collected, and the amount of cytokines released from tissue was measured using a customized Milliplex inflammatory cytokine immunoassay kit (Millipore, Burlington, MA, USA). Then, 96-well plates with filter bottom were used for the assays, according to the manufacturer’s recommendations. First, beads coated with primary antibodies against following cytokines were incubated with the sample medium: interleukin (IL)-10, IL-6, IL-1β, IL-2, IL-4, vascular endothelial growth factor (VEGF), IL-12p 70, regulated on activation, normal T cell expressed and secreted (RANTES), tumor necrosis factor (TNF)-α, and macrophage inflammatory protein (MIP)-1α. After the unbound sample was washed away, attached particles were bound again by a primary detection antibody conjugated with biotin, after which streptavidin-R-Phycoerythrin (Life Technologies) was used to detect the analyte. Fluorescent tag intensity was measured on Luminex 200™ System (Luminex) and analyzed with Magpix instrumentation software. Concentrations of individual cytokines were calculated from a logistic standard curve generated by measuring seven standard concentrations.

### 2.6. Behavioral Test

Locomotor recovery 7 days after SCI was evaluated in rats treated with pp242 (*n* = 5), RAPA (*n* = 5), and vehicle (*n* = 12) using the Basso, Beattie, and Bresnahan (BBB) open-field locomotor test [42]. Two independent examiners evaluated rats’ locomotor abilities by monitoring their movement in an open-field area for 4 min. Each animal received a BBB score (0–21) describing their ability to perform actions such as hindlimb joint movement, bodyweight support, forelimb–hindlimb coordination, and tail movement.

### 2.7. Statistical Analysis

To determine statistically significant differences between multiple treatment groups in all experiments, one-way ANOVA was used, followed by a Student–Newman–Keuls (SNK) post hoc test (Sigmastat 3.1, Systat Software Inc., San Jose, CA, USA). Differences were regarded as significant at *p* < 0.05. Data were plotted using GraphPad Prism version 5.0.0 for Windows (San Diego, CA, USA) as means ± standard error of the mean, while the level of statistical significance was marked as follows: * *p* < 0.05, ** *p* < 0.01, *** *p* < 0.001.

## 3. Results

### 3.1. Effect of RAPA and pp242 on the Recovery of SCI by Inhibition of mTOR Pathway

**Figure 1:**
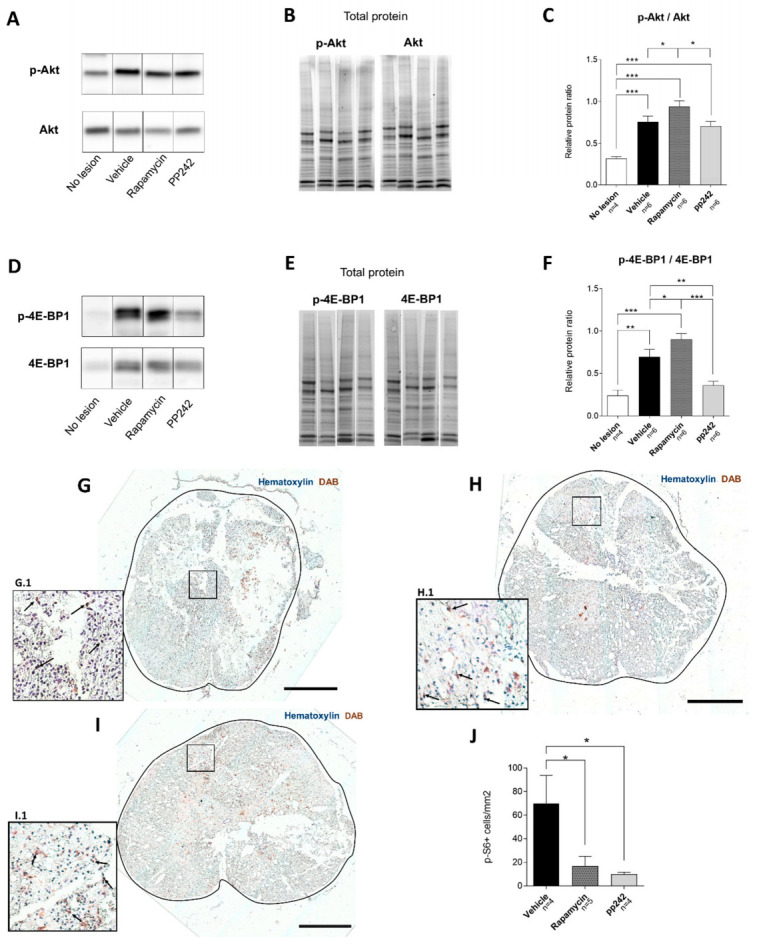
Suppression of mTOR pathway by RAPA or pp242 following SCI. Western blot analysis of proteins isolated proximal to the lesion site (**A**–**F**). Representative immunoblots of experimental and control groups are shown for each analyzed protein (**A**,**D**). Stain-free technology was used for visualization of total protein content (**B**,**E**) to ensure equal protein loading and normalization of relative band intensities of individual proteins. Representative total protein stains correspond to the sample lanes in representative blots of individual analyzed proteins. The phosphorylation level of proteins was assessed by calculating ratios of normalized phosphorylated and unphosphorylated protein band intensities. All rats after SCI had a higher p-Akt/Akt ratio compared to uninjured rats (**A**–**C**), and RAPA caused an increase in p-Akt/Akt (**C**). All rats after SCI had a higher p-4E-BP1/4E-BP1 ratio compared to uninjured rats (**D**–**F**), and RAPA caused an increase, while pp242 showed a comparable phosphorylation rate to that measured in healthy tissue (**F**). Immunohistochemical staining of spinal cord sections with phospho-S6 ribosomal protein (p-S6) and hematoxylin (**G**–**J**). Sections from rats treated with RAPA (**H**) and pp242 (**I**) had a significantly lower number of p-S6 positive cells per section area (**J**) compared to vehicle-treated controls (**G**). Scale bars: 500 µm; G.1, H.1, and I.1 images are 1:4 magnifications of corresponding areas of the spinal cord. Arrows point to examples of DAB-stained cells. Data are shown as means ± SEM; * *p* < 0.05, ** *p* < 0.01, *** *p* < 0.001.

The effect of mTOR inhibition in injured rat spinal cords was assessed by phosphorylation of its downstream effectors Akt and 4E-BP1, using Western blot analysis (Figure 1A–F). Relative ratios of normalized band intensities of p-Akt/Akt showed that the highest phosphorylation of Akt (Ser473) was present in the spinal cord tissue of the RAPA-treated group, being significantly higher than observed in pp242- (*p* = 0.031) and vehicle-treated animals (*p* = 0.043) (Figure 1C). Control tissue from rats without lesions had a significantly lower rate of p-Akt levels as compared to the spinal cords where the injury was induced (*p* < 0.001).

pp242 treatment caused significantly lower expression of p-4E-BP1 compared to the vehicle control group (*p* = 0.002). Although its level was downregulated compared to the control group without SCI, it did not reach statistical significance. Relative phosphorylation of 4E-BP1 was upregulated in the RAPA-treated group as compared to the pp242 (*p* < 0.001) and vehicle groups (*p =* 0.043) (Figure 1F). Healthy non-lesioned tissue had a significantly lower level of p-4E-BP1 compared to vehicle-treated animals (*p* = 0.001), as well as those treated with RAPA (*p* < 0.001).

#### p-S6 Levels Are Downregulated by mTOR Inhibitors

Activity and inhibition of the mTOR pathway was demonstrated by immunohistochemical analysis of p-S6, a substrate of S6K1 (ribosomal S6 kinase 1) that is regulated by the downstream product of mTORC1. The number of p-S6-positive cells was counted on transverse spinal cord sections 7 days after SCI (Figure 1G–J). The counted number of stained cells per section-area was found to be statistically higher in the vehicle-treated control group (Figure 1G) compared to both the RAPA- (*p* = 0.025) (Figure 1H) and the pp242-treated groups (*p* = 0.018) (Figure 1I) in five sections (two rostral and two caudal) taken from the center of the lesion. No significant difference was found between RAPA- and pp242-treated animals. We found that sections taken from tissue distal to the lesion center, which was located at T8–T9, had similar p-S6 expression in all groups (data not shown). This indicates that an approximately 4 mm portion of spinal cord containing the lesioned tissue was affected by RAPA and pp242 treatment.

### 3.2. Inhibition of mTOR Pathway by RAPA or pp242 Enhances Autophagy in SCI

**Figure 2:**
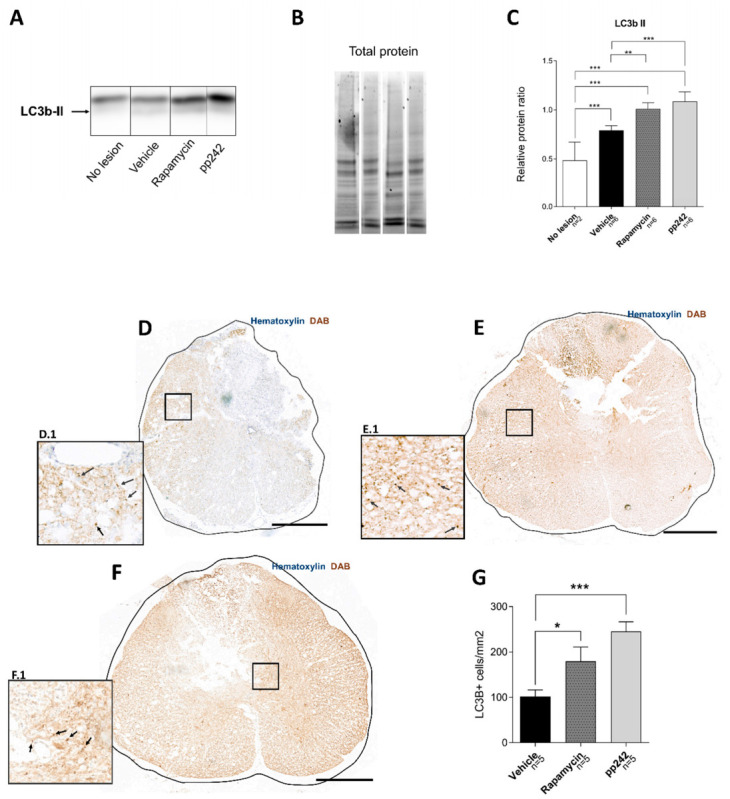
Separation of mTOR pathway by RAPA or pp242 enhances autophagy in SCI. LC3b-II content was assessed by Western blot analyses (**A**–**C**). Representative immunoblot lanes of each sample group are presented (**A**). Protein band intensities were normalized to total protein content visualized by stain-free technology (**B**). Representative total protein stains are corresponding to the sample lanes in representative immunoblots. LC3b-II levels were higher in all injured rats compared to non-injured rats; however, significantly higher levels were present in animals treated with RAPA or pp242 compared to vehicle-treated controls (**C**). Immunohistochemical analysis of LC3b in spinal cord sections from rats treated with vehicle control (**D**), RAPA (**E**), or pp242 (**F**) revealed a significant increase in LC3b expression in both RAPA- and pp242-treated groups (**G**). Scale bars: 500 µm; D.1, E.1, and F.1 images are 1:4 magnifications of corresponding areas of the spinal cord. Arrows point to examples of DAB-stained cells. Data are shown as means ± SEM; * *p* < 0.05, ** *p* < 0.01, *** *p* < 0.001.

Autophagy activation was investigated in spinal cord tissue after treatment with RAPA or pp242 in SCI. Levels of autophagy marker LC3b-II were measured using Western blot and immunohistochemistry (Figure 2). Western blot analysis (Figure 2A–C) showed an upregulation of LC3b-II after SCI in all treated groups compared to no-lesion controls (*p* < 0.001). Spinal cord tissue from vehicle-treated control rats showed significantly lower LC3b-II expression compared to RAPA- (*p* = 0.002) or pp242-treated rats (*p* < 0.001). However, no difference was seen in LC3b-II levels between RAPA- and pp242-treated groups (*p* = 0.228).

Immunohistochemical analysis of LC3b expression in spinal cord sections (Figure 2D–G) confirmed our Western blot results. An upregulation of the autophagy marker LC3b in pp242 (*p* < 0.001) and RAPA (*p* = 0.0254) was observed compared to vehicle controls. The increase in LC3b in pp242-treated animals was not significant when compared to RAPA-treated rats (*p* = 0.0513). LC3b marker expression was analyzed in sections located in the 4 mm spinal cord lesion core (T8–T9); however, we observed some upregulation in LC3b expression in treated groups even in sections located further from the lesion (data not shown).

### 3.3. Suppression of mTOR Pathway by RAPA or pp242 Alters Cytokine Production in SCI

**Figure 3:**
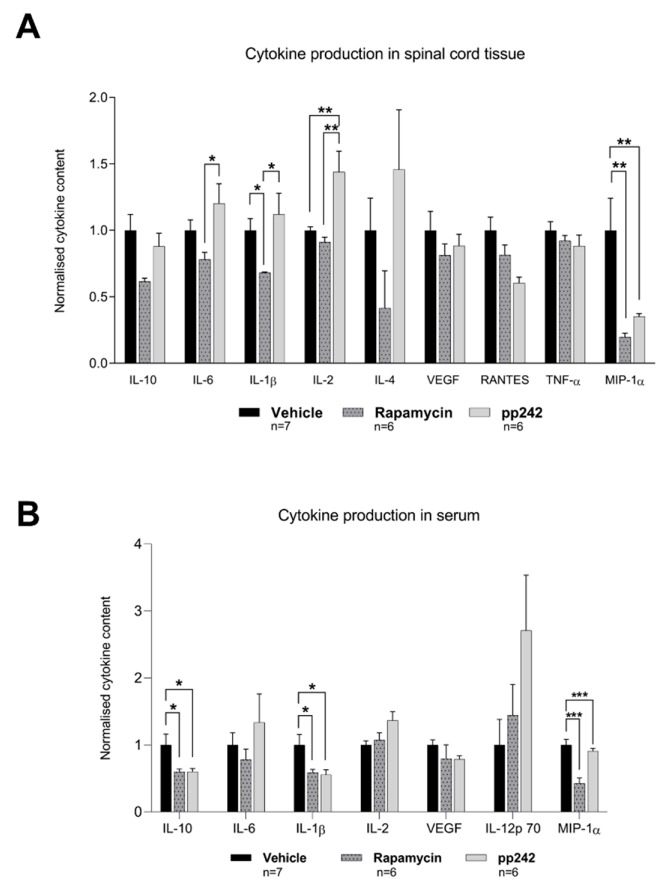
Suppression of mTOR pathway by RAPA or pp242 alters cytokine production in SCI. Analysis of cytokine levels in spinal cord tissue (**A**) and serum (**B**) after SCI. Calculated concentration data were normalized to the average concentration of the vehicle control group for each cytokine. Data are presented as means ± SEM; * *p* < 0.05; ** *p* < 0.01; *** *p* < 0.001.

To investigate the inflammatory response in treated rats after SCI, cytokine levels (IL-10, IL-6, IL-1β, IL-2, IL-4, VEGF, IL-12 p70, RANTES, TNF-α, and MIP-1α) in the spinal cord tissue and serum were measured (Figure 3). The RAPA-treated group had statically lower levels of IL-6 (*p* = 0.027), IL-1β (*p* = 0.026), and IL-2 (*p* = 0.002) in spinal cord tissue compared to the pp242-treated group, while contents of IL-1β (*p* = 0.044) and MIP-1α (*p* < 0.001) were lower compared to vehicle-treated controls (Figure 3A). pp242-treated animals had a significantly higher quantity of IL-2 (*p* = 0.003) and a lower MIP-1α (*p* = 0.003) content in tissue compared to the vehicle-treated control group.

Cytokine levels in rat serum revealed that RAPA- and pp242-treated animals showed significantly lower levels of IL-10 (*p* = 0.048 and *p* = 0.02), IL-1β (*p* = 0.019 and *p* = 0.03), and MIP-1α (*p* < 0.001 and *p* < 0.001), respectively, compared to saline-treated controls (Figure 3B). These findings of altered cytokine levels were not observed in our analyses of spinal cord tissue. In addition, the IL-2 level in serum was statistically higher in pp242-treated rats compared to vehicle-treated controls (*p* = 0.046).

### 3.4. mTOR Inhibition Leads to Structural and Functional Recovery in Acute SCI

**Figure 4:**
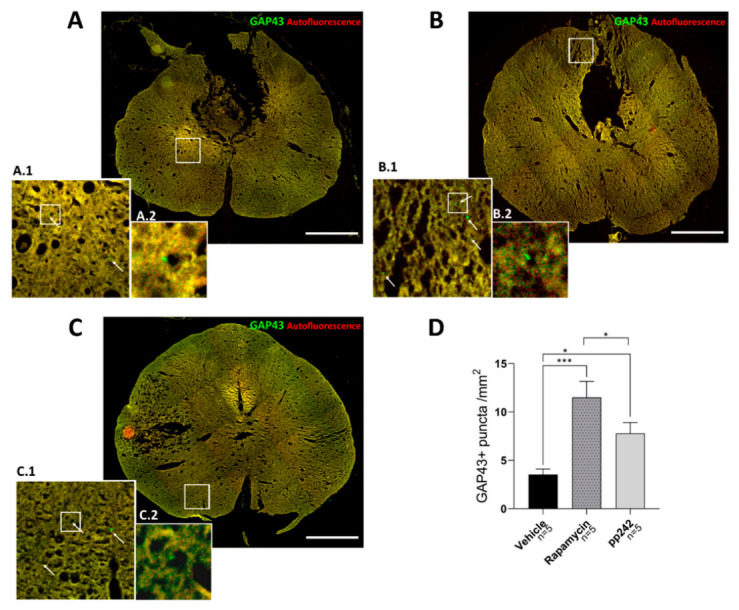
Inhibition of mTOR pathway by RAPA (**B**) leads to greater axonal sprouting assessed by the number of GAP43^+^ puncta compared to inhibition by pp242 (**C**) and controls (**A**), as shown in the graph (**D**). Scale bars: 500 µm; A.1, B.1, and C.1 images are 1:5 magnifications of corresponding areas of the spinal cord, whereas A.2, B.2, and C.2 are 1:4 magnifications of the indicated areas of A.1, B.1, and C.1, respectively. Arrows point to examples of GAP43^+^ puncta. Data are shown as means + SEM; * *p* < 0.05, *** *p* < 0.001.

Axonal sprouting was assessed by counting GAP43^+^ puncta in transversal spinal cord sections (Figure 4). To precisely visualize the puncta and exclude autofluorescence, sections were captured on both green and red fluorescence filter. Green GAP43^+^ puncta could then be recognized against the yellow background. Compared to the control group, both RAPA and pp242 groups had a higher number of GAP43^+^ puncta (*p* < 0.001 and *p* = 0.032). Interestingly, significantly lower axonal sprouting was observed in the pp242 group, compared to the RAPA-treated group (*p* = 0.016).

**Figure 5:**
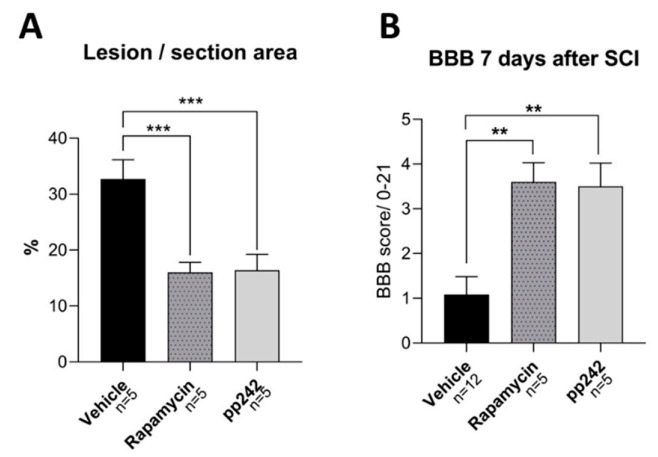
mTOR inhibition caused an improvement in locomotor function and a reduction in lesion size in acute SCI. A reduction in lesion size (**A**) was observed in the RAPA and pp242 groups. Significantly higher Basso, Beattie, and Bresnahan (BBB) scores (**B**) were observed following the treatment with rapamycin or pp242 after 7 days of SCI. Data are shown as means ± SEM; ** *p* < 0.01, *** *p* < 0.001.

Structural recovery of the spinal cord was also assessed by analysis of lesion size. Lesion size was measured in spinal cord sections and related to the area of spared tissue (Figure 5A, Figure A2). We found that lesion size in control group animals was larger compared to both RAPA- and pp242-treated rats (*p* < 0.001). These results suggest that suppression of the mTOR pathway by RAPA and pp242 mitigates the development of secondary injury and, thus, may promote recovery after SCI.

Behavioral locomotor recovery after SCI was examined using the BBB open-field test (Figure 5B). Both pp242 and RAPA treatment caused an improvement in BBB scores compared to vehicle-treated controls (*p* = 0.002 and *p* = 0.001). In the control group, we saw no or very limited movement of the hindlimbs in one or two joints 7 days after SCI, resulting in a very low average BBB score of 1.45, similar to scores reported previously on the same SCI model [8]. RAPA- and pp242-treated animals, however, showed slight or extensive movement of two or three joints in the hindlimbs and scored, on average, 3.5 and 3.6 on the BBB scale, respectively. No statistical differences in BBB scores or lesion sizes in RAPA- and pp242-treated groups were found.

## 4. Discussion

The PI3K/Akt/mTOR pathway plays a vital role in a plethora of physiological mechanisms, such as transcription, translation, cytoskeletal organization, and autophagy [43,44]. Its significance has been demonstrated in various conditions of the central nervous system, including SCI [19,27,45,46,47,48]. The inhibition of mTOR by RAPA leads to neuroprotection, as well as reduced secondary tissue damage, following SCI as reported previously [19,27,49,50]. RAPA is a well-known inhibitor of the mTOR pathway that works via analogous binding to FKBP12, and it exclusively inhibits mTORC1 [32]. Sustained inhibition of mTORC1 with RAPA causes a break in negative feedback, leading to activation of the PI3K/Akt pathway through insulin receptor substrate-1 expression [51]. The activity of mTORC2, as judged by phosphorylation of Akt, is activated via intracellular signaling [52], various growth factors, and ribosomal activation. mTORC1 regulates protein translation and growth, while mTORC2 is involved in many other cellular functions including cellular migration. In order to investigate the mTOR suppression in SCI, it is important to inhibit both complexes [31]. Although research on the inhibition of both mTORC1 and mTORC2 after SCI is limited [53], recent studies have indicated that dual suppression can provide more effective protection against secondary tissue damage and may lead to improved motor recovery. Our results here demonstrate that the BBB score was significantly improved and lesion size was reduced with the dual inhibitor pp242 compared to controls, but not compared to RAPA treatment (Figure 5). However, we are aware that the full effect of RAPA or pp242 treatment on functional outcome will only become apparent after a longer period of animal survival (6–8 weeks), and further experiments are required.

**Figure 6:**
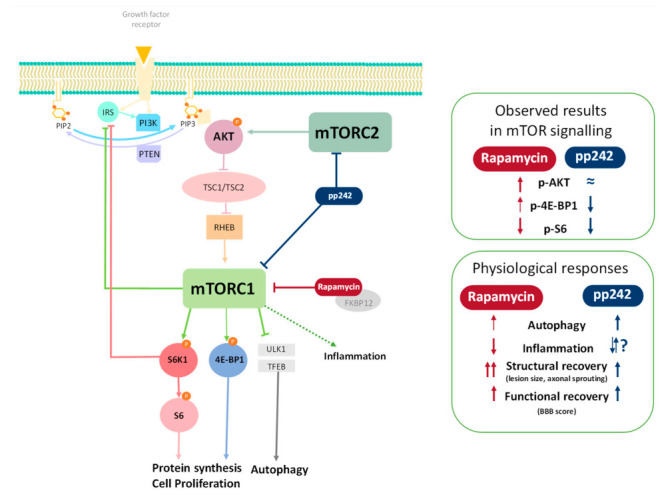
mTOR pathway and its inhibition by rapamycin (RAPA) and pp242 after spinal cord injury (SCI). RAPA, attached to FKBP12, inhibits mTOR complex 1 (mTORC1), which leads to decreased phosphorylation of downstream proteins S6, eukaryotic translation initiation factor 4E-binding protein 1 (4E-BP1), autophagy activation through ULK1 and transcription factor EB (TFEB), and reduced inflammation. A negative feedback loop through disinhibition of insulin receptor substrate-1 (IRS) is also induced with RAPA inhibition, which leads to activation of AKT, through phosphatidylinositol 4,5-bisphosphate (PIP2) conversion to phosphatidylinositol (3,4,5)-trisphosphate (PIP3) by phosphoinositide 3-kinase (PI3K). Akt inhibits tuberous sclerosis complex (TSC), which leads to disinhibition of the small GTPase RHEB (Ras homolog–mTORC1 binding) and RHEB activates mTORC1. Inhibition of mTORC1 and mTOR complex 2 (mTORC2) by pp242 leads to inhibition of AKT and, therefore, more effective inhibition of the mTOR pathway. Panels on the right display observed changes in protein modifications and physiological responses during inhibition of the mTOR pathway after SCI compared to vehicle treated controls. Phosphatase and tensin homolog (PTEN); ribosomal protein S6 kinase 1 (S6K1).

Our study demonstrated how RAPA and pp242 treatments altered the levels of substrates of the mTOR pathway from spinal cord tissue after acute SCI (Figure 6). A downregulation of p-S6 immunoreactivity in the spinal cord sections isolated from rats treated with RAPA or pp242 was seen (Figure 1). This result demonstrates that mTORC1 was successfully inhibited by RAPA and pp242, as suppression in mTORC1 activity leads to the inhibition of its substrate S6K1 phosphorylation, which in turn reduces phosphorylation of p-S6 [54]. Another substrate of mTORC1 is protein 4E-BP1 [55], whose phosphorylation state was upregulated in RAPA and vehicle-treated rats compared to the non-lesioned rats (Figure 1F). Successful inhibition of 4E-BP1 phosphorylation was provided only by pp242 treatment. The significance of these results remains to be understood. Some of these observations can be explained by previous studies reporting that p-4E-BP1 production, which can be resistant to RAPA treatment, is more effectively blocked by pp242 [56,57]. Jiang et al. [58] reported that RAPA treatment could cause resistance to 4E-BP1 phosphorylation, as shown in regenerating rat livers. Furthermore, RAPA has been shown to produce a differential regulation of S6K1 in comparison with 4E-BP1 in various cell lines [59]. In that sense, RAPA caused a stable inhibition of S6K1 phosphorylation and initially reduced 4E-BP1 phosphorylation. Prolonged treatment, however, leads to 4E-BP1 hyperphosphorylation, causing 4E-BP1 to dissociate from eIF4E, and it leads to a recovery in cap-dependent translation despite persistent S6K1 inhibition by rapamycin. This is consistent with our observation showing persisted dephosphorylation of p-S6 accompanied by hyperphosphorylation of 4E-BP1 after 5 days of RAPA treatment (Figure 1). In fact, mechanisms regulating such association/dissociation of mTOR-related proteins or posttranslational modifications on mTORC1 are suggested to exist, which can explain this phenomenon of differential phosphorylation [55].

We observed a noticeable increase in p-4E-BP1 expression, as a result of further activation of mTORC1 signaling and its substrates, leading to increased protein synthesis (Figure 1F). It is important to note that components of translation machinery are, in part, regulated by mTOR, including the regulation of the recruitment of ribosomes to mRNA [21]. Protein synthesis plays a critical role in both injury signaling [60,61,62] and the formation of new growth cones during regeneration. However, recent studies have suggested that an efficient supply of growth-promoting machinery to the axon is crucial for axon regeneration in CNS, and this appears to be missing from nonregenerative axons in the adult CNS [63]. Since the growth-promoting machinery needed for axon regeneration was lacking in our model of SCI, the suppression of pS6 expression by RAPA and pp242 at the lesion site appeared not to worsen the recovery from SCI, as it led to axonal sprouting (Figure 4). This result corroborates a previous finding, where axonogenesis and neuronal survival were observed after SCI treated with RAPA [64]. Rapamycin, an allosteric inhibitor of mTOR, may partially inhibit protein synthesis depending on the substrates [65]. Active-site/competitive inhibitors of mTOR, such as pp242, totally inhibit mTORC1 function to completely reduce protein synthesis rates in proliferating cells in vitro [66], which could be the reason why we saw less axonal sprouting in pp242-treated animals compared to RAPA.

Furthermore, we observed that the phosphorylation ratio of p-Akt/Akt was significantly higher in vehicle-treated controls as compared to no-lesion controls (Figure 1C). Such a pattern was sustained in RAPA- and pp242-treated rats. However, RAPA-treated rats had significantly higher phosphorylation of Akt relative to vehicle-treated controls. These findings of increased p-Akt in RAPA-treated rats are consistent with the findings of Li et al. showing a similar pattern [27]. It is possible that acute SCI led to a sustained increase in the mTORC2 pathway that was unable to be inhibited by these compounds. Unexpectedly, we also found that the p-Akt production rate in pp242 group was lower compared to vehicle controls. Such a discrepancy could be attributed to the fact that semiquantitative Western blot analysis was done on proteins isolated from an approximately 1 cm spinal cord tissue section, proximal to the lesion site. In fact, the immunoanalysis of p-S6 revealed that differences in expression between treated and untreated rats could only be detected in the immediate 4 mm vicinity of the center of the lesion (data not shown). Our immunoanalysis findings did not fully corroborate our Western blot analysis; regardless, these results together indicate that inhibition of the mTOR pathway by pp242 results in different downstream activity of the cascade in tissue after SCI compared to its inhibition by RAPA.

SCI is a complex pathophysiologic process that is influenced by numerous molecular mechanisms. In recent years, autophagy has been intensively studied in connection to secondary injury after SCI. It was confirmed by multiple studies that autophagic activity increased at the lesion site after SCI [16,20,27,45,67]. Autophagy has a beneficial role in SCI, and the accumulation of autophagosomes probably reflects the inhibition of autophagic flux and disruption of lysosomal functions [17]. The mTOR pathway has been recognized as a master regulator of autophagy [68], and its inhibition not only induces autophagy but also enhances lysosomal biogenesis [69]. Treatment of SCI with RAPA demonstrating favorable outcomes has been attributed to its ability to induce autophagy [48,49,50]. Dual mTOR complex inhibitors, including pp242, also induce autophagy and are more potent than RAPA alone [70]. However, their use in the treatment of SCI has not yet been demonstrated. Our findings showed that the LC3 b-II protein levels were elevated after SCI, and treatment with RAPA or pp242 further potentiated this increase in protein expression (Figure 2). Upregulation of the LC3b-II level in pp242-treated rats was higher compared to the RAPA-treated rats according to Western blot, as well as LC3b in immunohistological analysis. Our results strengthen the finding that mTORC1 activation suppresses autophagy, and that suppression of this complex with RAPA enhances the autophagic responses. Dual inhibition by pp242 did potentiate a further autophagic response, but not significantly. Therefore, the role of mTORC2 in autophagy is probably not substantial. This aspect of mTROC2 remains poorly understood, but it was proposed that mTORC2 indirectly suppresses autophagy by activating mTORC1 [71]. Autophagy induction leads to microtubule stabilization and promotes axon growth [72], which could explain why we saw increased axonal sprouting in pp242- and RAPA-treated animals (Figure 4).

Inflammation after trauma, marked by activation of microglia and astrocytes, infiltration of macrophages and neutrophils, and upregulation of proinflammatory cytokines, including IL-1β, IL-6, and TNF-α, are known contributors to neuronal loss during the secondary phase of SCI [3]. A reduction in the initial inflammation after SCI could reduce the magnitude of neuronal injury and consequent disability [8,9]. RAPA is a known immunosuppressant and has also been shown to suppress inflammation in experimental autoimmune encephalomyelitis, an animal model of multiple sclerosis [73]. SCI treatment with RAPA led to a reduction in TNF-α [49], while dual inhibition of mTOR was shown to also decrease the expression of IL-1β and TNF-α after SCI [53]. Our study demonstrated a decrease in IL-1β and MIP-1α cytokine levels in spinal cord tissue following RAPA treatment as compared to the vehicle-treated controls. Our results pertain to the studies showing that IL-1β is a proinflammatory cytokine, which contributes to neuronal damage and reduces locomotor recovery in the rat SCI model [74], while its absence positively affects neural outcome after SCI [75]. In addition, MIP-1α was shown to contribute to progressive tissue damage after SCI, while its absence resulted in increased locomotor recovery, smaller lesion size, and reduced neuronal damage after SCI [76]. Therefore, our findings that MIP-1α levels were suppressed following pp242 treatment may contribute to the improved recovery from SCI (Figure 5B).

Our additional findings showed that the levels of IL-6, IL-1β, and IL-2 were higher in pp242-treated animals compared to those treated with RAPA. IL-6 is a cytokine with multiple physiological roles, including inflammation, immunity, and regulation of cell differentiation. It was reported that upregulation of this cytokine is induced by SCI [77] and it appears to be detrimental, as multiple studies demonstrated that inhibiting IL-6 signaling with a neutralizing antibody improves locomotor recovery after SCI [78,79]. On the other hand, there has recently been accumulating evidence suggesting that IL-6 also has a role in structural and functional recovery following SCI [80]. Furthermore, IL-2 is essential in immunoregulation, as it regulates autoimmunity via the production of CD4^+^CD25^+^ T regulatory (Treg) cells. Administration of IL-2C prolongs the half-life of IL-2 and leads to upregulation of Treg cells in the ipsilateral brain cortex 3 days after traumatic brain injury [81]. Furthermore, treatment with IL-2/IL-2AB decreased brain infarction and enhanced neurological outcomes in a rat model of stroke [82]. Although these studies shed light on the involvement of IL-2 and Treg cells in the CNS, their role in SCI remains to be elucidated [83], as some studies regard IL-2 in SCI as proinflammatory [84,85] and others as anti-inflammatory [86]. Suppression of the mTORC1 complex by RAPA caused an anti-inflammatory response, with suppressed inflammatory cytokine production (IL-1β, MIP-1α) in the spinal cord. On the other hand, the dual inhibitor of the mTOR complex, pp242, led to an increase in proinflammatory cytokines. The latter finding remains to be explained, as treatment with pp242 had a favorable response in reducing mTOR activity and improving functional outcome and axonal sprouting, albeit to a lesser extent compared to RAPA (Figure 4). Despite these results, the functional recovery achieved by pp242 may not be related to the inflammatory response in the acute phase of SCI. Regarding systemic cytokine levels, RAPA and pp242 treatment resulted in generally similar serum cytokine production. Both treatments induced downregulation of inflammatory cytokines IL-1β and MIP-1α and lowered production of IL-10, an anti-inflammatory and neuroprotective cytokine [87].

## 5. Conclusions

In conclusion, the present study assessed the mechanisms via two mTOR pathway inhibitors, RAPA and pp242, which influenced the acute phase of SCI in rats. Our results showed that the intraperitoneal treatment with RAPA or pp242 caused distinct inhibition of mTOR pathway in the spinal cord tissue after SCI. In addition, similar upregulation of autophagy in spinal cord tissue, systemic inflammatory response, and functional recovery in the acute phase of SCI were achieved by RAPA and pp242 treatments. Several cytokines usually regarded as proinflammatory were found to be elevated in the spinal cord tissue of pp242-treated animals. Furthermore, RAPA was more effective in inducing axonal sprouting compared to pp242. In summary, our results suggest that treatment with pp242 did not produce a more effective response than RAPA in the treatment of acute SCI. We propose that benefits of mTOR inhibition in SCI treatment are mainly mediated through mTORC1. Additional inhibition of mTORC2 did not further amend outcomes of acute SCI, but its exact role in SCI should be explored further, for example, by selective mTORC2 inhibition.

## Figures and Tables

**Table 1:** Experimental groups of rats used in the study.

Experimental Group	Procedure	Treatment	Transcardial Perfusion with 4% Paraformaldehyde	Freshly Isolated Tissues	Total
RAPA	SCI	Rapamycin 5 mg/kg	5	6	11
pp242	SCI	pp242 5 mg/kg	5	6	11
Vehicle	SCI	Saline, DMSO, triton	5	7	12
No lesion	n/a	n/a	n/a	2	2

## Data Availability

The data presented in this study are available on request from the corresponding author.
